# High prevalence of typhoidal *Salmonella enterica* serovars excreting food handlers in Karachi-Pakistan: a probable factor for regional typhoid endemicity

**DOI:** 10.1186/s41043-015-0037-6

**Published:** 2015-12-08

**Authors:** Taranum Ruba Siddiqui, Safia Bibi, Muhammad Ayaz Mustufa, Sobiya Mohiuddin Ayaz, Adnan Khan

**Affiliations:** 1 Gastroenterology and Hepatology unit, Pakistan Medical Research Council, Research Center, Jinnah Postgraduate Medical Center, Refiquee Shaheed Road, Karachi, 75510 Pakistan; 2 Pakistan Medical Research Council, Research Center, National Institute of Child Health, Karachi, Pakistan; 3 Microbiology Department, University of Karachi, Karachi, Pakistan

**Keywords:** Typhoid fever, Diarrhea, Endemics, *Salmonella enterica* serovars, Carriers

## Abstract

**Background:**

Typhoid fever is the persistent cause of morbidity worldwide. *Salmonella enterica* serovar’s carriers among food handlers have the potential to disseminate this infection on large scale in the community. The purpose of this study was to determine the prevalence of typhoidal *S. enterica serovars* among food handlers of Karachi.

**Methods:**

This cross-sectional study was conducted in Karachi metropolis. A total of 220 food handlers were recruited on the basis of inclusion criteria from famous food streets of randomly selected five towns of Karachi. Three consecutive stool samples were collected from each food handler in Carry Blair transport media. Culture, biochemical identification, serotyping, and antimicrobial susceptibility tests for *S. enterica* serovars were done.

**Results:**

Out of 220 food handlers, 209 consented to participate, and among them, 19 (9.1 %) were positive for *S. enterica* serovars. Serotyping of these isolates showed that 9 (4.3 %) were typhoidal *S.* serovars while 10 (4.7 %) were non-typhoidal *S.* serovars. Of the typhoidal *S.* serovars, 7 were *S. enterica* serovar Typhi and 1 each of *S. enterica* serovar Paratyphi A and B. The resistance pattern of these isolates showed that 77.7 % were resistant to ampicillin and 11.1 % to cotrimoxazole. All typhoidal *S. enterica* serovar isolates were sensitive to chloramphenicol, ceftriaxone, cefixime, nalidixic acid, and ofloxacin.

**Conclusions:**

Carrier rate of typhoidal *S. enterica* serovars in food handlers working in different food streets of Karachi is very high. These food handlers might be contributing to the high endemicity of typhoid fever in Karachi, Pakistan.

## Background

Typhoid fever remains a public health problem worldwide. It is caused by *Salmonella enterica* serovar Typhi and *S. enterica* serovar Paratyphi A, Paratyphi B, and Paratyphi C. A recent study on global burden of typhoid fever reported 26.9 million illnesses and 200,000 to 600,000 deaths annually due to typhoid fever [1]. Typhoid is endemic in most of the developing countries like Pakistan. A prospective population-based surveillance conducted in five Asian countries including Pakistan revealed that the annual typhoid incidence is the second highest, i.e., 412.9 (per 100,000 person years), in Pakistan [2]. A study conducted in Pakistan in pediatric population also reported 170/100,000 incidence of typhoid fever annually [3]. This high incidence of typhoid fever in Pakistan is mainly contributed by persistent poverty, poor personal hygiene, and sanitary condition [4].

Typhoid fever can be cured with appropriate antimicrobial treatment still 3–5 % of patients become lifelong carriers [5]. Since the organism is transmitted through fecal oral route, hence, these carriers serve as a main source for the transmission of infection as they continue to harbor and excrete the organism in their feces. Carriers of these pathogens among food handlers may be another reason for endemicity in these areas as they transmit the infection on a large scale in the community. Worldwide it is recommended that in case of food handlers, microbiological clearance of cases, carriers, and contact cases should be performed. At least five consecutive negative sets of cultures should be done to ensure safe food handling [6].

Although many studies have been previously conducted in Pakistan on typhoid incidence, antimicrobial resistance in *Salmonella* and its serovars, we could not find any data regarding prevalence of *Salmonella* carrier in our population particularly in food handlers [3, 7–9]. Taking into account the poor condition of sanitation, hygiene, and no guidelines for safe food handling in this highly endemic area, estimation of *Salmonella* carrier state particularly in food handlers is of the utmost importance. The aim of this paper is to describe the estimate of *Salmonella* carrier state in food handlers working in different regions of Karachi. Evidence from this paper is useful to do intervention-based study which leads to the formulation of guidelines for safe food handling in our setup. Keeping in view the high resistance rate in *S. enterica* serovars and emerging multiple drug resistance from previous study [2], the antimicrobial susceptibility pattern of *Salmonella* isolated from food handlers’ stool samples is also assessed in this study.

## Methods

### Study setting

**Fig. 1:**
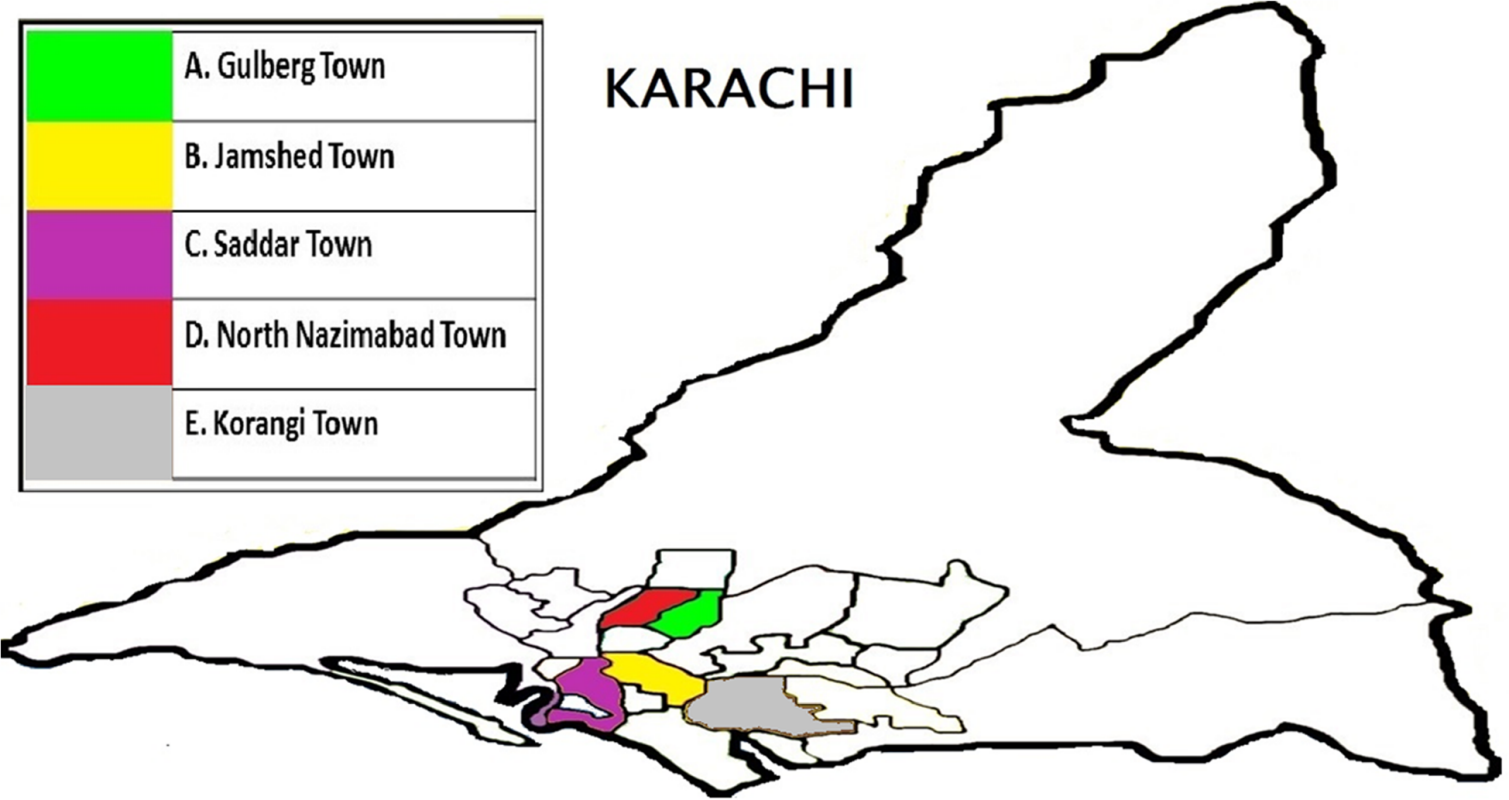
Map of Karachi Metropolis, Sindh, Pakistan, showing towns distribution. Study towns are colored (Karachi and towns maps are adopted from the official website of Metropolis Karachi) http://www.kmc.gos.pk/

**Fig. 2:**
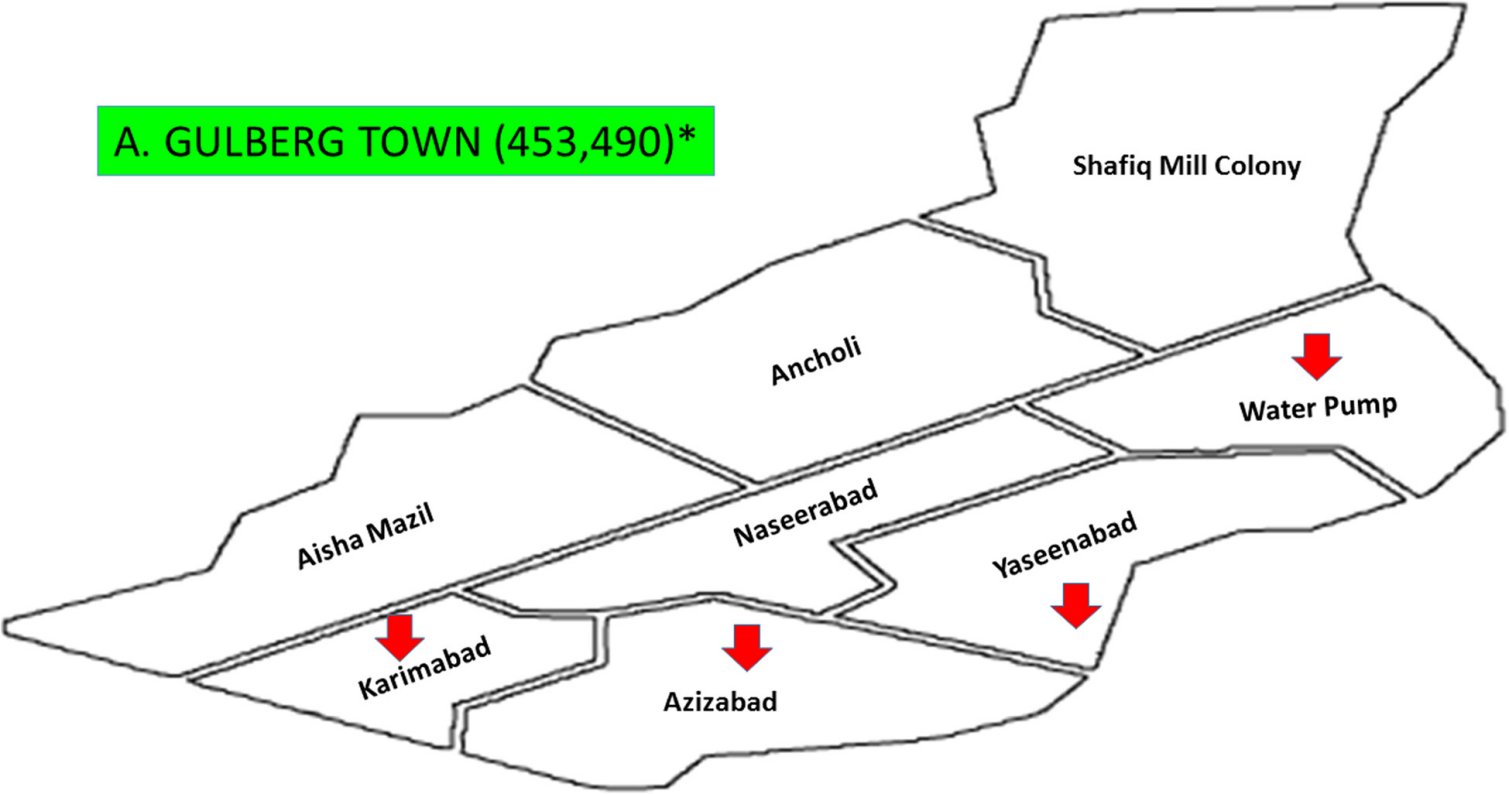
Gulberg Town showing with arrow signs pointing to food streets area visited in this study for data collection. Asterisk showing population of this town  according to the last census 1998. (Karachi and towns map is adopted from the official website of Metropolis Karachi) http://www.kmc.gos.pk/

**Fig. 3:**
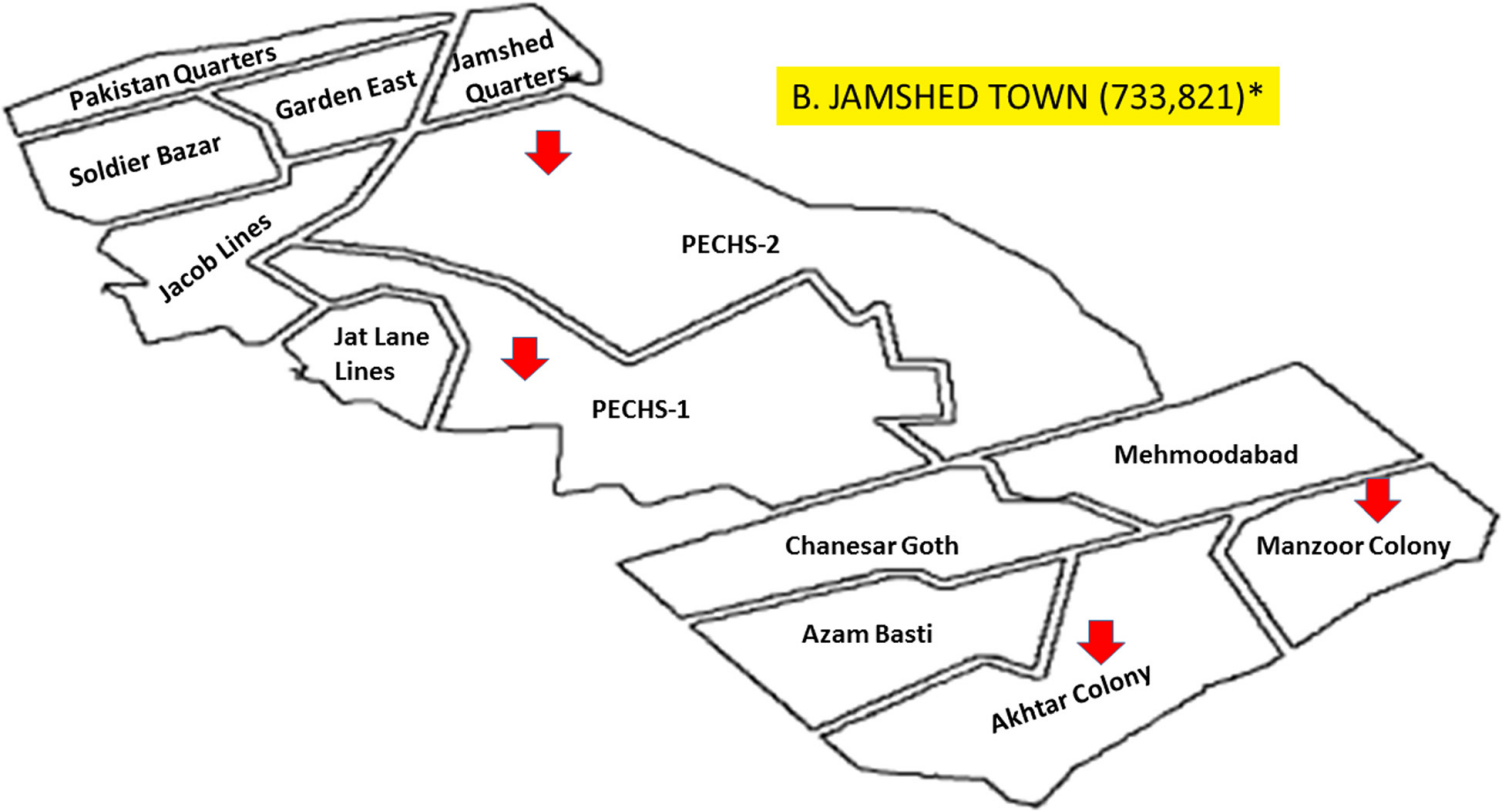
Jamshed Town showing with *arrow signs* pointing to food streets area visited in this study for data collection. *Asterisk* showing population of this town according to the last census 1998. (Karachi and towns map is adopted from the official website of Metropolis Karachi) http://www.kmc.gos.pk/

**Fig. 4:**
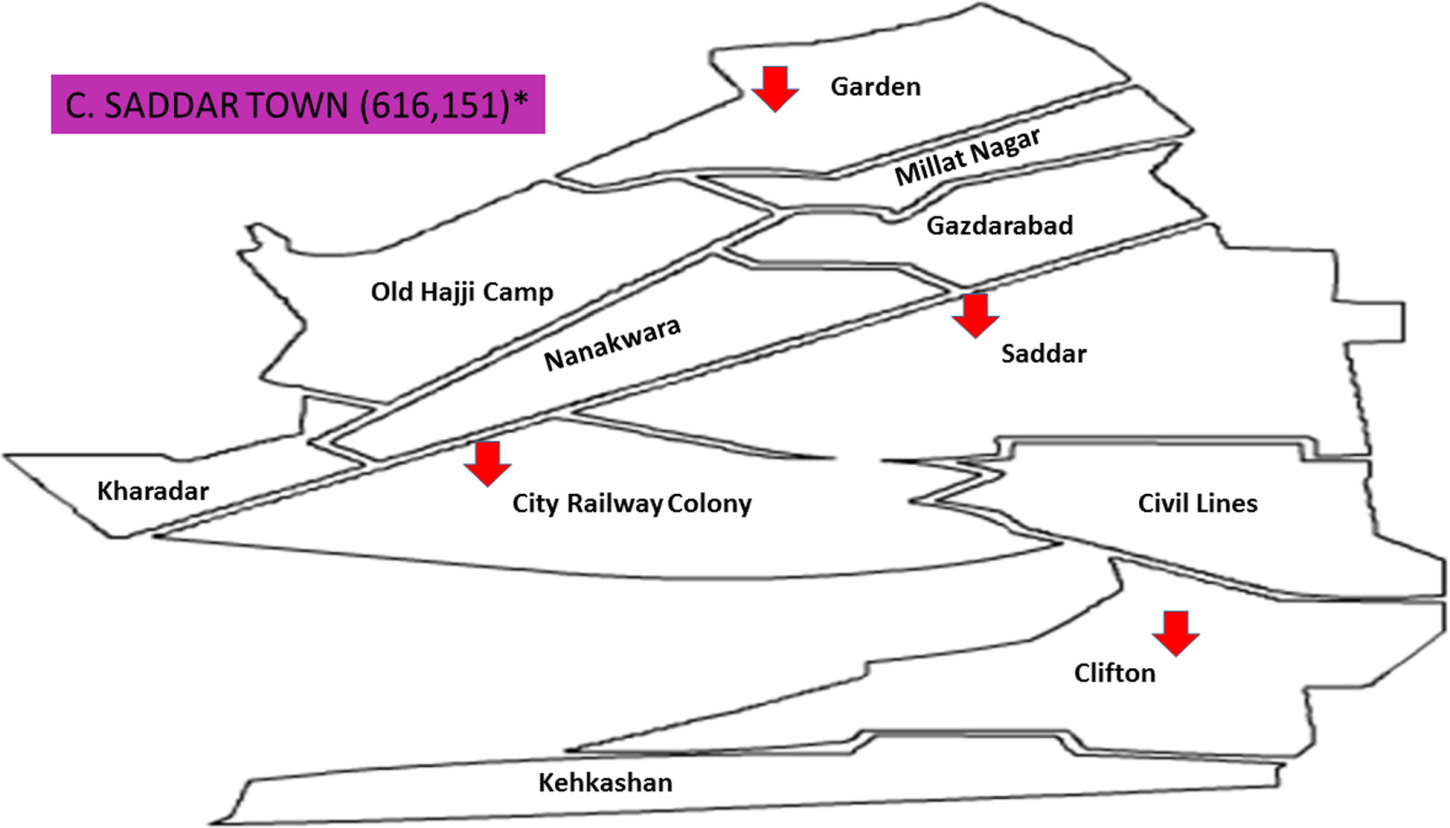
Saddar Town showing with arrow signs pointing to food streets area visited in this study for data collection. *Asterisk* showing population of this town according to the last census 1998. (Karachi and towns map is adopted from the official website of Metropolis Karachi) http://www.kmc.gos.pk/

**Fig. 5:**
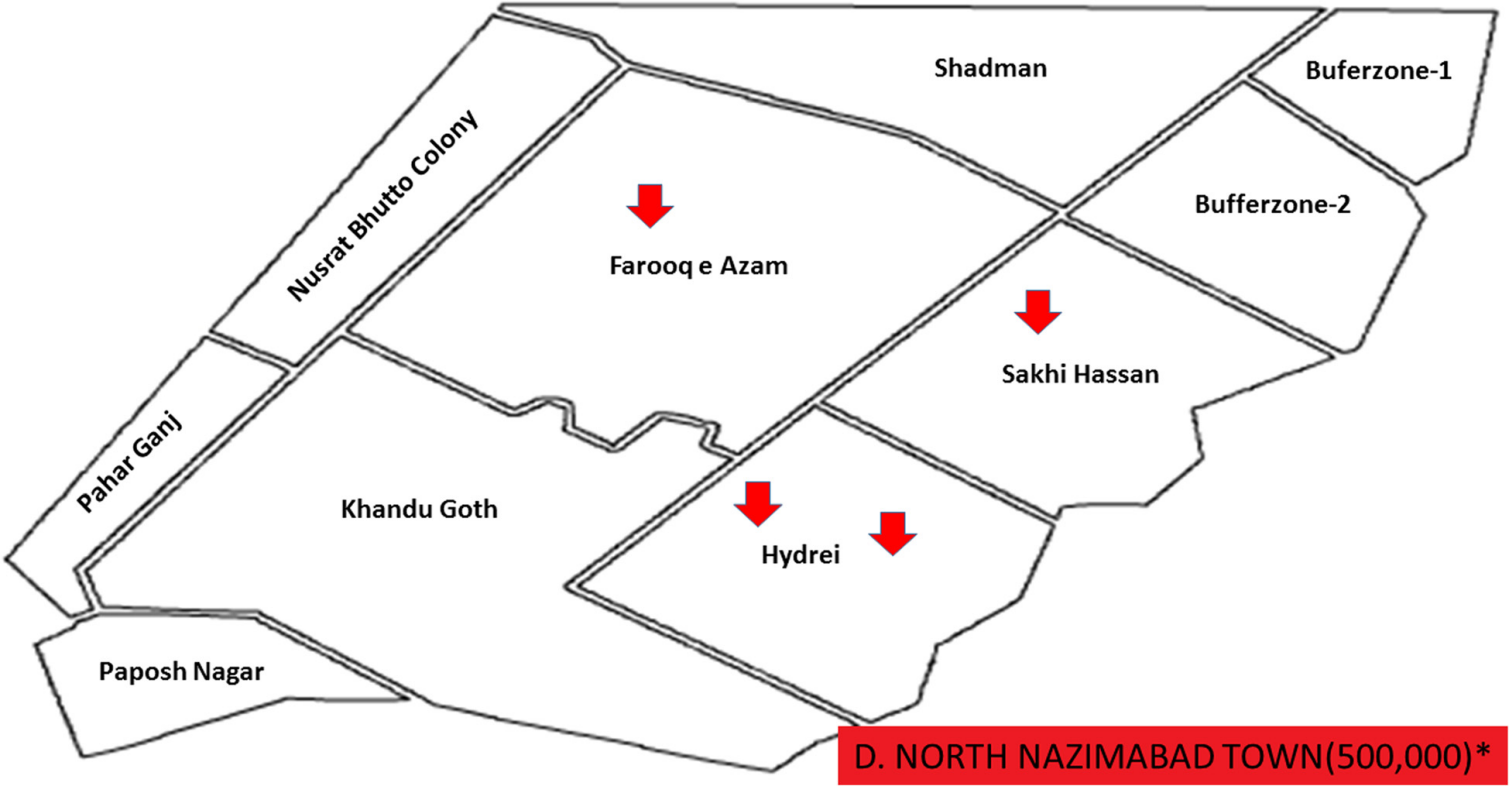
North Nazimabad Town showing with arrow signs pointing to food streets area visited in this study for data collection. *Asterisk* showing population of this town according to the last census 1998. (Karachi and towns map is adopted from the official website of Metropolis Karachi) http://www.kmc.gos.pk/

**Fig. 6:**
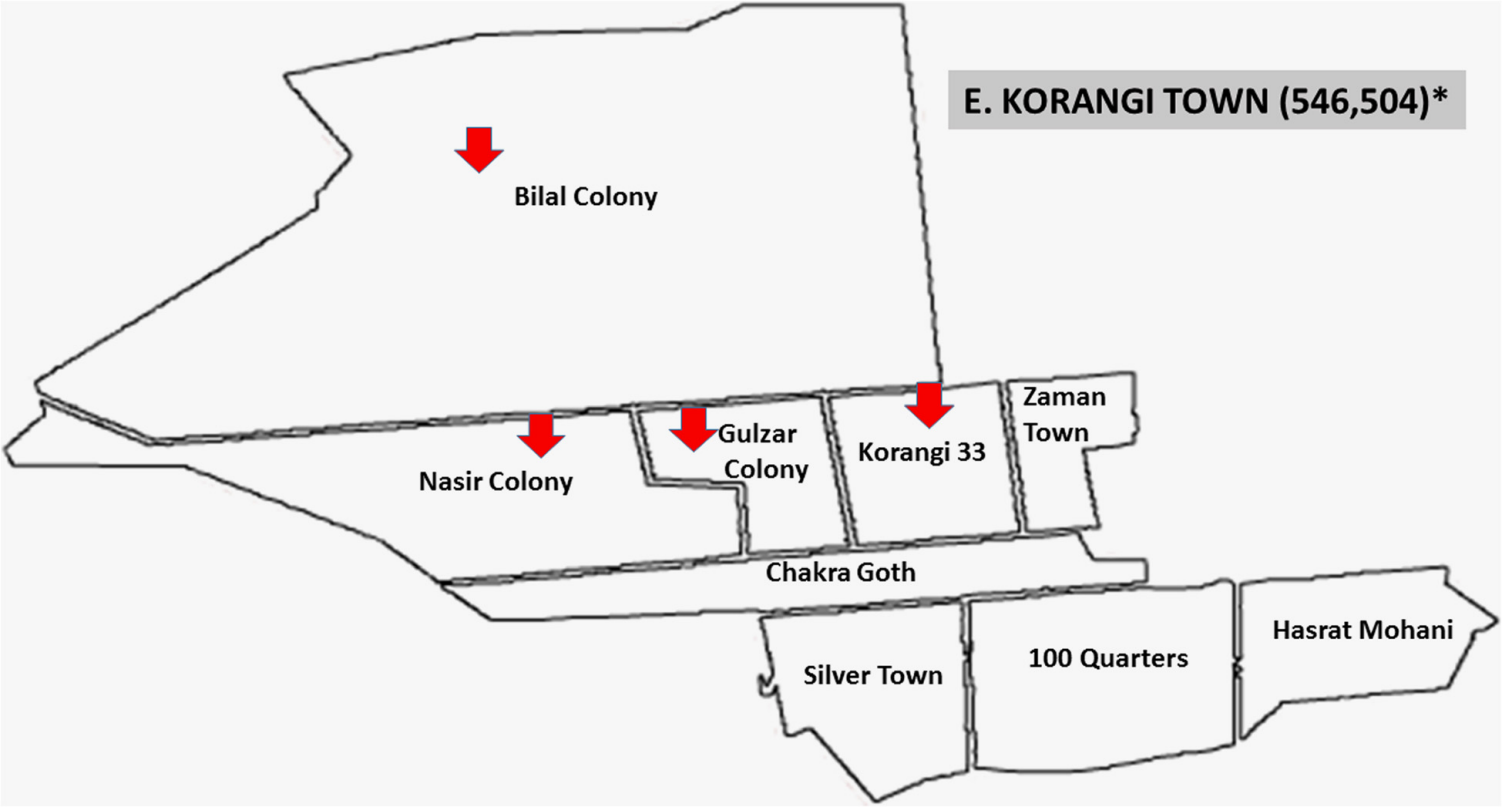
Korangi Town showing with arrow signs pointing to food streets area visited in this study for data collection. *Asterisk* showing population of this town according to the last census 1998. (Karachi and towns map is adopted from the official website of Metropolis Karachi) http://www.kmc.gos.pk/

It was a cross-sectional study in which from eighteen towns of Karachi, five towns, named as Gulberg Town, Jamsheed Town, Sardar Town, North Nazimabad Town, and Korangi Town, were randomly selected. From each selected towns, four food streets were recruited for the study (Figs. 1, 2, 3, 4, 5, and 6). These selected food streets were visited to approach food handlers for interview and stool samples.

### Sample size

On the basis of previous study [10] with 7 % precision and the design effect (D_eff_) of 2 at the 95 % confidence level, the sample size for this study was calculated as 220 food handlers (with 5 % extra due to noncompliance).

### Study population

Food handler was defined as “A person involved in the preparation, cooking, serving or transportation of food in any part of the institute or hotel or restaurant” [11].

Apparently, healthy food handlers were recruited. Those who have recalled past 3 months illness with high-grade fever (>38 °C) and diarrhea or recently confirm typhoid cases in food handlers were excluded from the study.

### Sampling

Food handlers from selected food street were approached for demographic information and stool samples. The purpose of the study was explained, and written informed consent was obtained from participants. Through pre-tested structured questionnaire, food handlers were interviewed regarding typhoid risk factors and history of typhoid fever. They were briefed about stool sample collection procedure through verbal and a written description. They were given a sterile stool container and Cary-Blair transport media with a cotton swab in air-tight plastic bags. Stool samples of each food handler were collected for three consecutive days and transported to Microbiology Laboratory of “Pakistan Medical Research Council, Research Center; Jinnah Postgraduate Medical Center,” Karachi Pakistan.

### Laboratory procedure

Culture and sensitivity test was performed according to the Clinical and Laboratory Standards Institute 2012 (CLSI) guidelines in Microbiology Diagnostic and Research laboratory of “Pakistan Medical Research Council, Research Center, JPMC.” Three differential and selective culture media, i.e., Xylose-Lysine Deoxycholate agar, Cysteine F broth and *Salmonella Shigella* Agar, were used for the isolation of *Salmonella* from stool samples. Biochemical identification of pathogens was performed on the bases of oxidase test, sulfide production, motility test, indole test, urea test, and reaction on triple sugar iron (TSI). For further identification of *Salmonella* serovars, serotyping was done by slide agglutination test (as per kit recommendation for each antigen). Polyvalent “O,” *Salmonella* Factor 2, 4, and 9, and Vi *Salmonella* antisera (Remel, UK) were used to confirm *S. enterica* serovar Typhi and *S. enterica* serovar Paratyphi A and Paratyphi B.

Antimicrobial susceptibility test (AST) was done by Kirby-Bauer disk diffusion method (Clinical Laboratory Standards Institute 2012) against ampicillin, chloramphenicol, ceftriaxone, nalidixic acid, ofloxacin, cotrimoxazole, cefixime, and ciprofloxacin.

### Follow-up

Food handlers were revisited to communicate their stool test reports, information about proper hygienic practices emphasizing on the importance of hand hygiene while handling foods were conveyed to them. Food handlers who were positive for *Salmonella* were referred to “Specialized Centre of Gastroenterology and Hepatology unit, PMRC, JPMC” for treatment and counseling in order to maintain strict hygienic practices while handling food.

### Ethics

The study was approved by the Institutional Review board (IRB) of Jinnah Postgraduate Medical Center. Written informed consents were taken from all the study participants prior to the data collection.

### Statistical analysis

Data analysis was done on computer software SPSS version 17.0 and Microscoft Excel. The chi-square test is used to check the possible association of typhoidal *Salmonella* isolates with risk factors. *p* value <0.05 was consider significant.

## Results

Out of 220 food handlers, 209 consented to participate and gave three consecutive stool samples. Their ages ranged from 13–70 years (Mean ± SD 30 ± 10.9). Only four food handlers were females. Of the total, 88 (42.1 %) were working as cook, 54 (25.8 %) as helper (those who helps in kitchen and serving food), and 67 (32 %) as steward.

Piped water for household and drinking was available to 171 (82 %) of food handlers while 61 (29 %) were treating their drinking water prior to use. Carrier cases and source of drinking water showed no significant association with carrier state.

*Salmonella* carrier rate was higher in food handler who belonged to middle socioeconomic group (*p* value 0.029). Hand washing before taking meal was practiced by 181 (86.6 %) of food handlers. Hand washing was infrequently practiced in *Salmonella* carriers as compared to noncarrier food handlers (*p* value 0.005). No significant association was observed between eating habit and carrier state. Among food handlers, 121 (58 %) food handlers used soap for hand washing. Consumption of fruits and vegetable without washing with carrier state in food handlers showed a significant association (*p* value 0.002).

A total of 38 (18 %) food handlers had the history of typhoid fever, and 15 (39.4 %) of them were hospitalized due to typhoid fever. In food handlers who were carriers of *Salmonella*, only 5 (55.5 %) had history of typhoid fever due to which 2 (22.2 %) were hospitalized. Significant association was observed between carrier state and history of typhoid fever in food handlers (*p* value 0.011). Family members of *Salmonella* carriers 3 (33.3 %) had history of typhoid fever. The detailed finding regarding hygienic practices and history of typhoid fever is given in Tables 1 and 2.

**Table 1:** Risk factors in *Salmonella* carriers

	Risk factors	*Salmonella* carrier		*p* value
		(Typhoidal + non-typhoidal)		
		Yes (*n* = 19)	No (*n* = 190)	
1	Socioeconomic status			
	Low	4 (21 %)	90 (47.4 %)	0.029
	Middle	15 (78.9 %)	90 (47.4 %)	
	High	0	10 (5.3 %)	
2	Washing of hands before taking meal			
	Always	12 (63.15 %)	169 (88.9 %)	0.005
	Never	7 (36.8 %)	21 (11 %)	
3	Eating habit			
	Always eat food cooked at home	9 (47.4 %)	79 (41.6 %)	0.400
	Eat from small restaurant (Thelas/Chapra hotels)	10 (52.6 %)	111 (58.4 %)	
4	Use of soap for washing			
	Always	13 (68.4 %)	108 (56.8 %)	0.234
	Sometime	6 (31.6 %)	82 (43.1 %)	
5	Washing of fruits and vegetables before consumption			
	Always	4 (21 %)	108 (56.8 %)	0.002
	Sometimes/never	15 (78.9 %)	82 (43 %)

**Table 2:** Typhoid history in typhoidal *Salmonella* carriers

History of typhoid fever		Typhoidal (*n* = 09)	Non-typhoidal and not carrier (*n* = 200{10 + 190})	*p* value
1	History of typhoid fever in food handlers			0.011
	Yes	5 (55.5 %)	33 (16.5 %)	
	No	4′(44.4)	167 (83.5 %)	
2	Typhoid treatment taken by food handler			0.104
	Yes	3 (15.8 %)	25 (13.2 %)	
	No	6 (66.6 %)	175 (87.5 %)	
3	Hospital admission for typhoid fever			0.129
	Yes	2 (22.2 %)	13 (6.5 %)	
	No	7 (77.7 %)	187 (93.5 %)	
4	Family member had typhoid fever			0.056
	Yes	3 (15.8 %)	19 (10.0 %)	
	No	6 (66.6 %)	181 (90.0 %)	

Total of 19 (9.1 %) food handlers were positive for *S. enterica* serovars while 9 (4.3 %) isolates were typhoidal *S. enterica* serovars. Seven (3.3 %) isolates were *S. enterica* serovar Typhi, 1 each (0.47 %) was *S. enterica* serovar Paratyphi A and B. Out of 19 isolates, 10 (4.7 %) isolates were non-typhoidal *S. enterica* serovars.

**Fig. 7:**
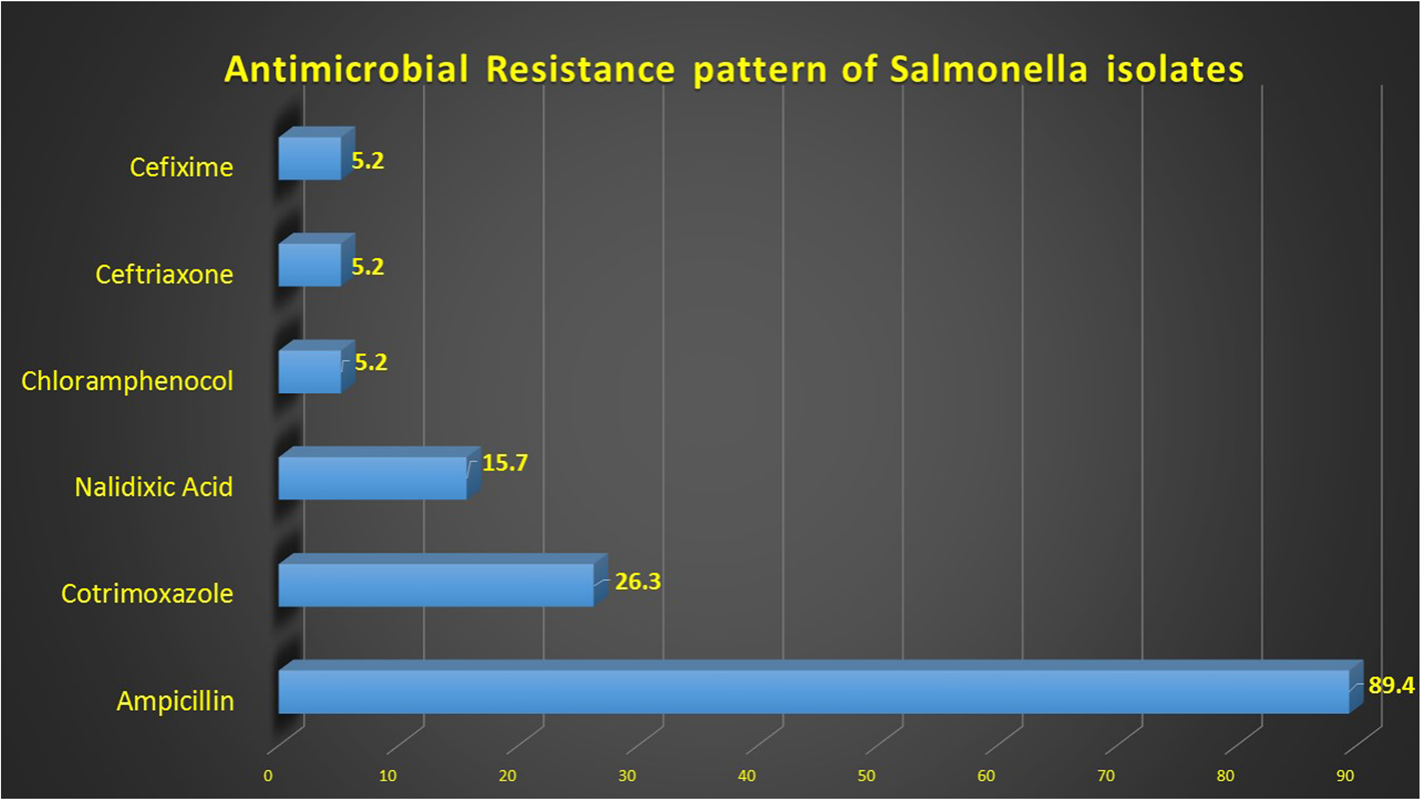
Figure showing Antimicrobial Resistance pattern of *Salmonella* isolates

Susceptibility pattern of typhoidal *S. enterica* serovars showed that 77.7 % were resistant to ampicillin and 11.1 % to cotrimoxazole. All typhoidal *S. enterica* serovar isolates were sensitive to chloramphenicol, ceftriaxone, cefixime, nalidixic acid, and ofloxacin. Non-typhoidal *S. enterica* serovar was 100 % resistance to ampicillin, 40 % to cotrimoxazole, 30 % to nalidixic acid, and 10 % each to chloramphenicol, ceftrixone, and cefixime. All isolates were sensitive to ofloxacin (Fig. 7).

Out of 19 food handlers who carried *Salmonella,* 9 had typhoidal serovars; of them, 3 were cooks, 2 were stewards, and 4 were helpers, while 10 food handlers were positive for non-typhoidal serovars were 4 cooks, 4 stewards, and 2 helpers.

## Discussion

Present study showed that 4.3 % healthy food handlers were carriers of typhoidal *S. enterica* serovars which is much higher than the study reported from Iran, i.e., 1.88 % [12]. Another study conducted in India showed 16.6 % carriers’ rate which is comparably very high [13]. We may correlate this dissimilarity in carrier rate with a population based surveillance data, showing higher incidence rate of typhoid, i.e., 493.5/100,000 in India followed by 412.9/100,000 in Pakistan [2].

The overall carrier rate of *Salmonella* serovars was 9 %. This is similar to studies from China [14] and UK [15] where 9.5 and 12.3 % were carriers of *S. enterica* serovars. However, this rate is much higher as compared to the studies carried out in Ethiopia and Ghana [16, 17]. Fecal carriage of non-typhoidal *Salmonella* in asymptomatic food handlers is 4.7 % which is higher as compared to study from Ghana which reported 1.1 % carriers of non-typhoidal *Salmonella* among food handlers [17].

Previous study showed that multidrug resistance (MDR) *S. enterica* is increasing [18] and varying geographically [2]. In present study, 77.7 % isolates showed resistance to ampicillin and 11 % showed resistance to cotrimoxazole; it is observed that none of the isolates was resistant against chloramphenicol, cephalosporins, and quinolone group. No typhoidal *Salmonella* MDR strain was isolated in the study which is comparable with the study from Nepal showing no MDR isolates [19], but contrast to the local hospital-based study which showed 30.5 % resistance to all three first line drugs in typhoidal S*almonella* serovars isolated from typhoid cases [20]. Another local study [21] also stated 30 % resistance strains against nalidixic acid which is used as a marker to detect intermediate/reduced susceptibility of ciprofloxacin against *S. enterica* serovars Typhi [22]. In the present study, none of the typhoidal *Salmonella* isolate showed resistance against nalidixic acid.

Out of 4.7 % non-typhoidal *Salmonella* carriers, 10 % MDR strains were isolated. Since salmonellosis (non-typhoidal) is a food-borne disease caused by consumption of contaminated foods and also contracted through fecal-oral route. In the existence of poor hygienic condition, this high rate of MDR is an alarming situation for public health concerns.

Though the treatment for *Salmonella* carrier is cholecystectomy, as gall bladder is the reservoir for this disease, but being an invasive procedure gall bladder removal is not feasible. Present study showed that fluoroquinolone (ofloxacin/ciprofloxacin) can be the drug of choice for the treatment of these carriers. A previous study from neighboring country also stated ofloxacin as a better option in presence of MDR *S. enterica* [23].

Assessment of living behavior and life style in relation to typhoid carrier state of food handlers revealed that most of the *Salmonella* carriers were not practicing hand washing before taking meal. World Health Organization (WHO) recommends safe water access and hygienic food handling to prevent typhoid (www.who.int/water_sanitation_health/diseases/typhoid/en/). In Karachi, thousands of food handlers are working in different restaurants/hotels. Many of these restaurants are small and located in insanitary areas. Besides that a large number of street food vendors are also working in approximately all localities of Karachi, there is almost no provision of sink and toilet in case of street food vendors. Hand washing is an established way to prevent disease transmission, but this basic step which breaks the infection chain is not routinely performed by most of the food handlers of Karachi. Other factors like eating outside from restaurants or stalls, washing of vegetables and fruits before consumptions, and history of typhoid fever in food handlers or within their families showed contributing factors which might lead to *Salmonella* infection/carrying *Salmonella* in their feces. Present study showed significant association between *Salmonella* carrier state and hygienic practices like hand washing and washing of raw food before consumption. In present study, the prevalence of *Salmonella* carrier was significantly higher in lower and middle socioeconomic classes; it was found that none of the food handlers belonging to higher class were *Salmonella* carrier. Findings of food handlers’ behavior towards hygiene are comparable with the study conducted in Indonesia; these findings confirm that poor hygienic practices of lower and middle socioeconomic classes are risk factors of acquiring this disease [24].

## Conclusions

It may be concluded from the present study that the carrier rate of typhoidal *Salmonella* serovars in food handlers is quite high. In an environment of poor sanitation and hygiene, this high rate signifies wide dissemination of typhoid pathogen through food handlers and indicates one of the probable reasons of typhoid endemicity in Karachi.

### Recommendation

It is recommended that local food regularity authority should establish pre-employment screening and medical clearance of food handlers; food handlers should also be screened periodically for *Salmonella* carrier state. In order to evaluate food handlers’ practices and contamination level, it is suggested that level of personal hygiene practices during their work should also be assessed (hand swabbing and microbiological analysis can be the assessing tool). Food handlers need to be aware of the importance of personal health and hygiene for the safety of food. It is recommended that awareness program should be organized for food handlers to encourage and motivate them about good practices. These efforts might play an important role in reducing the burden of typhoid endemicity in Karachi Metropolis.
